# Relationship between judges’ scores and dive attributes from a video recording of a diving competition

**DOI:** 10.1371/journal.pone.0273374

**Published:** 2022-08-24

**Authors:** Bianca Luedeker, Monnie McGee

**Affiliations:** Department of Statistical Science, Southern Methodist University, Dallas, TX, United States of America; Universidade do Vale do Rio dos Sinos, BRAZIL

## Abstract

Sports such as diving, gymnastics, and ice skating rely on expert judges to score performance accurately. Human error and bias can affect the scores, sometimes leading to controversy, especially at high levels. Instant replay or recorded video can be used to assess judges’ scores, or sometimes update judges’ scores, during a competition. For diving in particular, judges are trained to look for certain characteristics of a dive, such as angle of entry, height of splash, and distance of the dive from the end of the board, to score each dive on a scale of 0 to 10, where a 0 is a failed dive and a 10 is a perfect dive. In an effort to obtain objective comparisons for judges’ scores, a diving meet was filmed and the video footage used to measure certain characteristics of each dive for each participant. The variables measured from the video were height of the dive at its apex, angle of entry into the water, and distance of the dive from the end of the board. The measured items were then used as explanatory variables in a regression model where the judge’s scores were the response. The measurements from the video are gathered to provide a gold standard that is specific to the athletic performances at the meet being judged, and supplement judges’ scores with synergistic quantitative and visual information. In this article we show, via a series of regression analyses, that certain aspects of an athlete’s performance measured from video after a meet provide similar information to the judges’ scores. The model was shown to fit the data well enough to warrant use of characteristics from video footage to supplement judges’ scores in future meets. In addition, we calibrated the results from the model against those of meets where the same divers competed to show that the measurement data ranks divers in approximately the same order as they were ranked in other meets, showing meet to meet consistency in measured data and judges’ scores. Eventually, our findings could lead to use of video footage to supplement judges’ scores in real time.

## Introduction

There are many competitive sports where expert judges score performance of participants, such as gymnastics [1], diving [2], figure skating [3], and power lifting [4]. In these sports, judges typically follow a set of guidelines in order to align their scores to a set of standards [4, 5]. However, it is impossible to eliminate all subjectivity when human judges are employed. In fact, there have been several scandals where judging bias was alleged in high-level competitions, particularly at the Olympics [2, 6, 7]. For example, fifteen out of seventeen judges who judged their own countrymen in the 2000 Olympics exhibited a nationalistic bias [8], and occasionally the amount of bias was enough to alter medal standings [2]. Furthermore, the bias extends beyond favoring athletes from the judge’s home country. It was also found that poorer athletes received higher scores than they deserved possibly due to sympathy [8]. Other types of bias occurring in subjective scores are reputation bias, where athletes with better reputations obtain higher scores [9]; serial position bias, where an athlete following a well-performing competitor gets higher scores than perhaps deserved [10]; and viewpoint bias [11], where errors in judging can be made depending on the judges’ view of the athlete. These papers all note that bias exists, but none suggest any method for adjusting scores for the bias or for reducing bias, other than providing more training and screening of judges. Mercier and Heiniger [12] suggest a method for evaluating judges, thus providing a score for integrity and accuracy for each judge. Their idea is to use their marking system for judges to reward the highest ranked judges with assignments to more prestigious events; thus elevating the entire judging system.

The main problem with determining the best judges, or conversely determining bias in judging, is a lack of a gold standard, other than the expertise of the judges themselves. By a gold standard, we mean a measure of the overall ability of an athlete to inform the fairness of scores for the current competition. Heiniger and Mercier [13] use the median scores of gymnasts from previous meets as a comparison to judges’ scores in an Olympic meet; however, this assumes that the athletes will perform similarly in the current meet as they have in the past. Injuries and miscalculations sometimes cause an athlete to perform less well than expected. Another article by the same authors concentrates on calculation of an evaluation metric for judges in international sports competitions [12]. The authors present an evaluation of judges in eight Olympic sports, of which diving is one. To measure ability of the athletes, they use a control score, which is the median panel score, on the grounds that it is less sensitive to outliers than the mean, and also a good approximation to true performance quality [13]. They also state that video analysis for some sports is available; however, [12] did not have access to the videos to use in their analysis; therefore, the videos were not used to calibrate judges’ scores.

Heiniger and Mercier [13] indicate the need for two things to ensure fairness in judging. The first necessity is a way of judging athlete ability that is independent of judges’ scores within a given competition. Even though any athlete can have a poor performance on a given day, in general, the best athletes are consistent in their performance. In order to examine judges’ score for accuracy, it is important to have some outside metric for comparison. The second necessity is subjective scores by expert judges, because automated scores for judged events are not possible [13]. However, for many events, video footage is available after the event, as evidenced by recaps using slow motion footage after each competitor has completed a round or an event [14, 15]. The video footage can be hard to access, and even harder to analyze, as it requires painstaking measurements from a human using specialized video editing software. Video has the advantage that it can be shot from multiple places in the arena, which means that a composite video ought to eliminate viewpoint bias [11]. In addition, video can allow measurements consistent with the set of standards that judges are using to generate their own scores, as shown for our study (see Results). With the advent of better video footage and computer software, it may be possible to supplement judged scores with video footage in real time.

Sports fans are familiar with the use of instant replay as a device for reviewing plays and calls in order to ensure the fairness of the game. Instant replay was controversial at first, but has become mainstream [16]. Advances in video technology have lead to the use of video for performance improvement in training [17], and also to view player behavior during team sports [18] in order to recall selected video instances from previously recorded athletic behaviors. The main objective for these video analyses is to examine game and player strategy *after* the game in order to inform performance and strategy in following games [15]. Other types of extant use of videos are videos of coach behavior, souvenir movies of international tournaments, and the occasional “blooper” movie for comedic purposes [16]. During the production of videos, it is important to be aware of trends in quantitative performance indicators in identifying relevant video sequences to display in order to capture the instances that are the most informative for future use [14].

Although video is prevalent, and some would say ubiquitous in sport, the focus of previous work has been on the analysis of video after a match or a meet [14, 15, 18]. Furthermore, most articles on the use of video in sport are focused on team sports, as a scan through the reference list at the end of this article indicates. Instant replay video is used in judged individual sports, such as gymnastics, diving, and ice skating, for reliving an amazing moment or reviewing a call for accuracy [16]. There is some precedence for combining the use of video in tandem with judges’ scores in international ice skating competitions. In ice skating, technical panels consisting of five specialists use videos of each skater’s performance to review and identify each element and the level of difficulty for that element [19]. The technical panel of judges serves as a gold standard for assuring elements are judged correctly.

We seek to provide a gold standard for diving meets that is specific to the meet being judged and supplemental to the judges’ scores, as mentioned in [13]. We accomplish this task by filming and measuring characteristics of divers during a meet using recorded video and image analysis software. Special software can integrate statistical information produced during analysis into videos to provide synergistic quantitative information and visual information [18]. In this article we show, via a series of regression analyses, that certain aspects of an athlete’s performance measured from video after a meet provide similar information to the judges’ scores themselves. Demonstration of the validity of certain aspect of video footage for judging a competition makes the use of supplemental video credible in judged competitions. The ultimate goal would be to use the video footage to supplement judges’ scores in real time.

## Materials and methods

### Data description

In January 2019, footage was collected from a regional high school diving meet using a Canon T-3i filming at 60 frames per second. Twenty-six divers, 14 female and 12 male, competed in eleven rounds of diving on a one-meter springboard. As is typical in club and high school diving meets, divers received scores ranging from 0 to 10 from five independent judges [20]. Scoring protocols, including the number of points to be awarded for each aspect of a dive, mandatory point deductions, and a skeletal scoring rubric, are given on pages 14–18 of [20]. In each round, the maximum and minimum judges’ ratings were discarded, and the remaining three scores were summed to get a round subtotal. The subtotal was multiplied by the degree of difficulty of the dive performed in that round to get the round total. The round totals were then summed and used to rank the divers to determine the winners in the men’s and women’s division [5].

Using recorded video, selected characteristics of each dive were measured. There are many important features of a dive that can contribute to the score such as height in the air, distance from the diving board, speed of rotation, compactness while in the air, angle of entry, and amount of splash. Due to time constraints and software limitations, only a few measurements from each dive could be collected. This subset included:

- Maximum height in the air: maximum vertical distance from the diver’s center of gravity to the surface of the water.
- Distance from the diving board: horizontal distance from the edge of the diving board to where the diver’s body enters the water.
- First angle of entry: the number of degrees past vertical of the diver’s body in the first three frames once the diver has broken the water’s surface
- Second angle of entry: the number of degrees past vertical of the diver’s body once the diver’s waist has entered the water.

**Fig 1:**
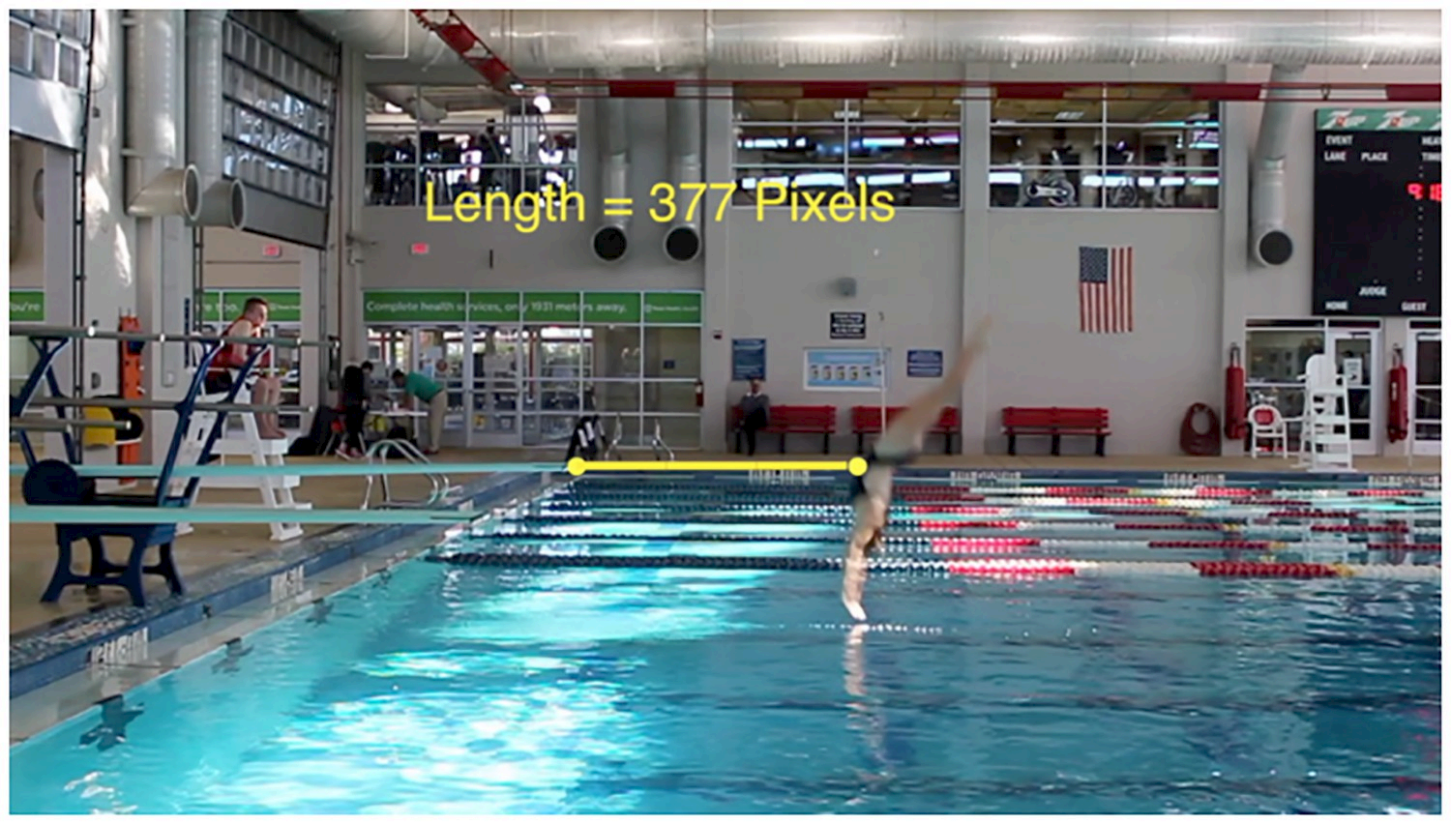
Measuring distance from the diving board at entry. Still photo illustrating how distance of the diver from the board was measured from the recorded footage.

**Fig 2:**
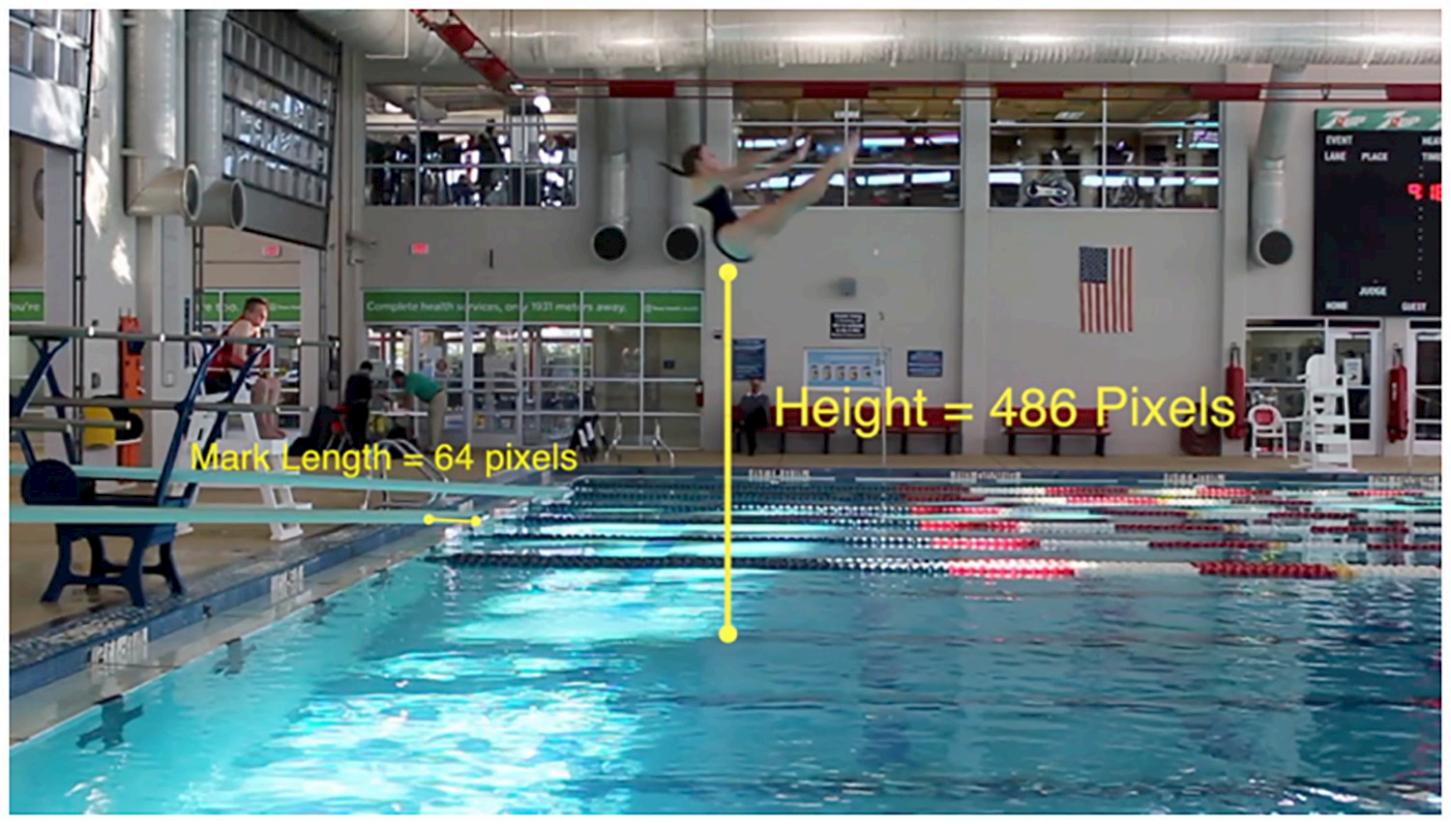
Measuring height of the dive. Still photo illustrating how height of the dive was measured from the recorded footage.

These measurements were collected manually using the film editing software Final Cut Pro X [21]. Measurements were taken by examining the pixels between a fixed object and the diver. For example, distance from the diving board was determined by placing pixels at the edge of the diving board and the spot on the water where the diver breaks the surface. The difference between the *x*-coordinates of the pixels was calculated. However, both the height measurement and distance measurement depended upon the tightness of the camera angle. We devised our own method for standardizing height and distance measurements due to camera setup. The black mark at the end of the diving board was measured in pixels; this distance was called the “mark length”. The height and distance measurements were then divided by the mark length. Hence, the units on height and distance are in terms of mark length. This allowed a consistent measurement among footage clips when the camera setup had changed. Pictures illustrating how these measurements were collected are in Figs 1 and 2.

**Fig 3:**
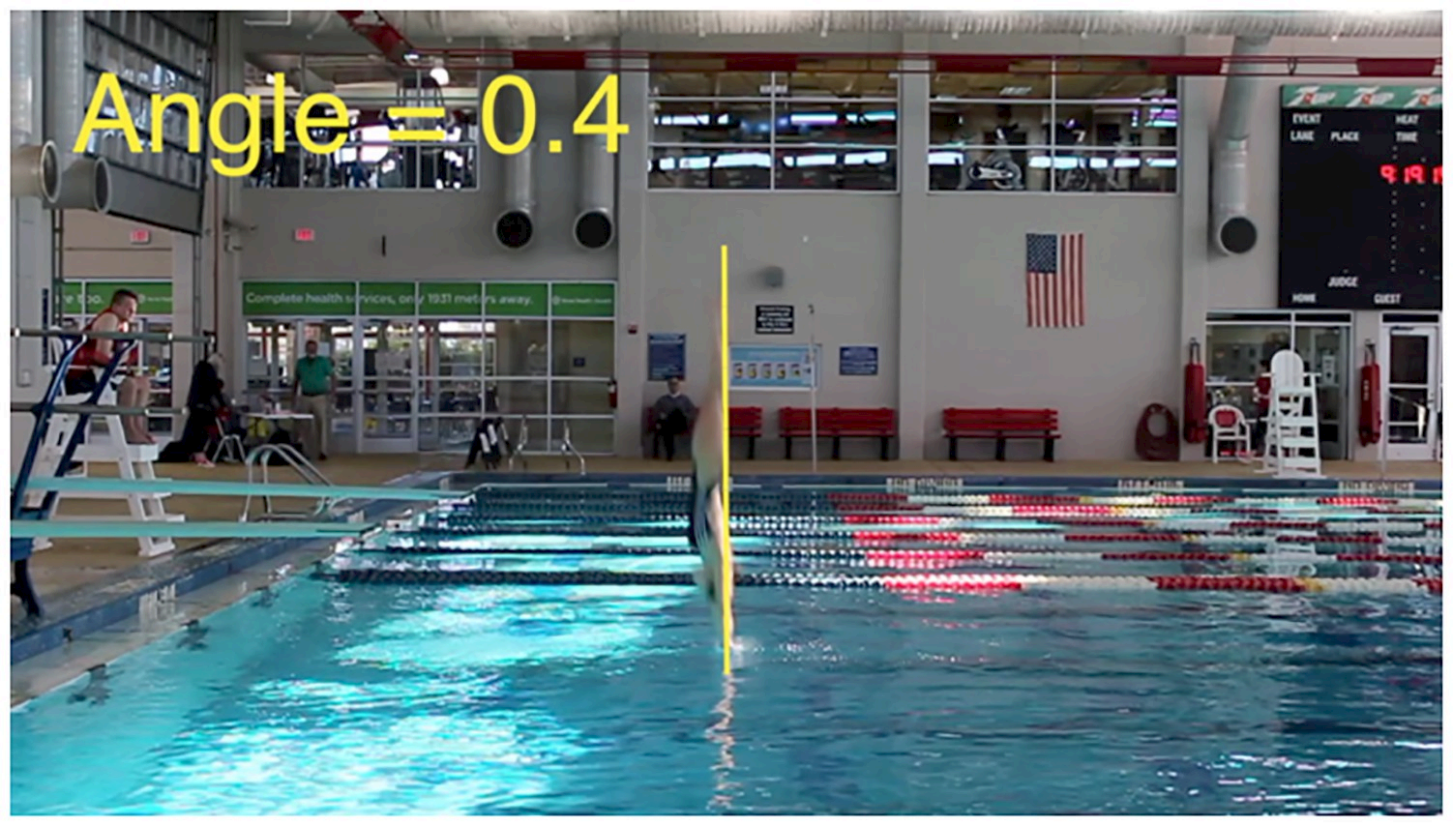
Measuring angle of dive entry. Still photo illustrating how angle of diver entry to the water was measured from the recorded footage.

Angle of entry was measured in a similar fashion. It was necessary to include two angle measurements because many divers would enter the water bent at the waist. Having the legs at an angle to the torso causes more splash, which negatively impacts judges’ scores. Using one angle measurement was not enough to accurately describe the diver’s body position. Angle measurements are given as the positive number of degrees past vertical; therefore, a small angle measurement means the diver entered the water nearly vertically. An illustration of the angle measurement is given in Fig 3.

In addition to the variables measured from the footage, there were four other explanatory variables recorded. These were gender of diver, degree of difficulty for the dive, position of the dive (tuck, pike, straight, free), and the round number (one through eleven). Scores for each dive were obtained by taking still photos of the scoreboard after each dive. This data was then manually entered into an Excel spreadsheet along with the characteristics measured from the footage.

All athletes participating in the meet are members of the Amateur Athletic Union (AAU), and written permission for filming was granted from the AAU [22]. Because the data collection involved only observation of public behavior without participation by the investigator in the activities being observed, the Institutional Review Board Committee of Southern Methodist University determined that the study is exempt from IRB oversight and informed consent under 45 CFR 46.101(b)(2). Given that the participants were displaying behavior where there is a reasonable expectation of public knowledge of the participants’ names and affiliations, and the observations were conducted in a public school–sponsored space, informed consent of the participants or their legal representatives is not required under section 46.104(d) of The Belmont Report [23]. Written parental permission was obtained via email for use of the photos of the divers in Figs 1–3. The data given in the supplemental material do not contain divers’ names, grades, and school affiliations, as these variables could easily lead to identification of the diver.

### Data cleaning

Missing data has been known to have a deleterious effect on measurement outcomes. Results based on complete case analysis, the subset of data that contains no missing values, are inefficient and biased [24]. A solution is to use some form of imputation to estimate the missing values before doing any analysis. van der Heijden, Donders, Stijnen, & Moons investigated the effects of missing data on multiple logistic regression models and found that the predictors selected for inclusion in the model differed depending on whether a complete case analysis was used or whether missing values were imputed [25]. They concluded that, for their data set, single imputation worked as well as multiple imputation and performed much better than complete case analysis [25].

We were unable to measure elements of seven out of 286 total dives because footage of these dives was not obtained. This was due to the camera overheating, a camera battery needing to be replaced, a SD card being full, or adjustment of the camera angle at the wrong time. None of these causes for missingness are related to the data values; therefore, the missing observations can be considered missing completely at random (MCAR). The missing data for these dives was imputed using the package hot.deck in the statistical software suite R [26]. We used the best cell single imputation method to impute missing values. In this method, rows with missing data are compared with rows that do not have any missing data; we will call these complete rows. Complete rows that are most similar to the missing data rows are used as donors. A sample of values of the missing variable are drawn from the donor rows where the probability of selecting a donor row is based on how similar it is to the missing data row [26].

There is undeniably measurement error in the data. Placing a pixel in the correct spot was sometimes difficult. Final Cut Pro X has a “snapping” function that will occasionally pull the pixel to a certain (*x*, *y*) location despite the best efforts of the video editor. This can make it impossible to place a pixel precisely. Another issue is that there is visual distortion that takes place when trying to represent the three dimensional world as a two dimensional moving picture. As we all know, in pictures, the distance between two parallel lines will decrease as the lines vanish toward the horizon. No adjustments were made to account for the visual distortions.

The goal of this project is to remove the subjectivity and bias that arises from human judges’ preferences and biases. However, using the footage, a human must still decide on the video frame in which to take measurements such as angle of entry and maximum height. In other words, making judgment calls when collecting measurements from the footage was unavoidable. Angle of entry, for example, is not fixed. It changes frame by frame for some divers. Choosing when to measure angle of entry was based somewhat on convenience. The choice was limited to no more than three frames, but the choice was subjective. The same was true about measuring height in the air. It is not always clear when a diver has reached the apex of his or her dive. Many of these problems could be remedied by using computer automation to measure the height and angle over multiple frames and taking an average over these measurements.

Information from 285 out of the 286 dives was used. One dive was removed from the data set because it was a failed dive and had a score of 0. Including failed dives greatly influences the coefficients and causes problems with the normality of the error terms. Furthermore, failed dives are decided by a consensus of all five judges [5]; therefore, the decision to fail a dive does not reflect independent judges’ assessments. Thus, the model presented in this paper should only be used to predict the score of completed dives.

Note that the set of judges’ scores for separate dives, *s*_*ij*_, where *i* represents the diver and *j* represents the judge, are not independent. The correlation structure is complex. All scores from judge *j* are correlated and all scores for diver *i* are correlated. Associated with each diver is a response matrix of size 11 by 5. In this paper, the underlying correlation structure is ignored in order to get a baseline regression model. In other words, we assume that each dive is independent of all other dives, including dives from the same diver. It should be noted that there is precedent for treating each dive as independent [2, 8]. In addition, each diver had to wait approximately 20 minutes between successive dives in order for all divers to complete a round; therefore, dives for the same diver could be considered independent given the time delay. In an attempt to reduce the complexity of the problem, we used the mean of the middle three judges’ scores as the response variable. This yields a one-dimensional response variable and mitigates the issue of independence of the scores assigned by judge *j*. In addition, the sum of the middle three judge’s score is what is used to obtain the round score for each diver [5]; therefore, use of the mean of the middle three judges’ is aligned with the meet scoring system.

## Results

**Fig 4:**
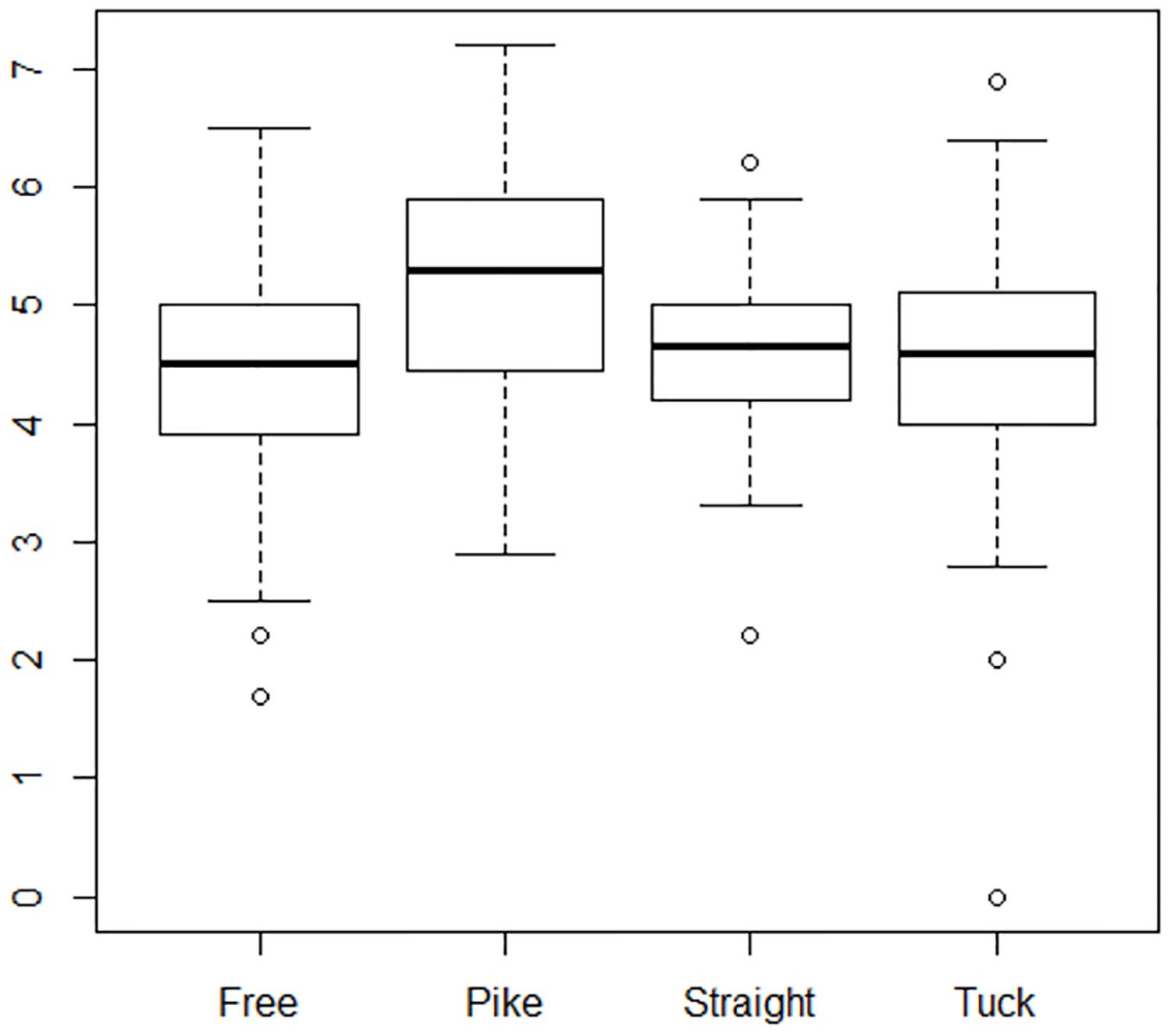
Distribution of mean scores for diving positions. Box plots showing the distribution of mean scores for all four possible dive positions: free, pike, straight, and tuck.

**Fig 5:**
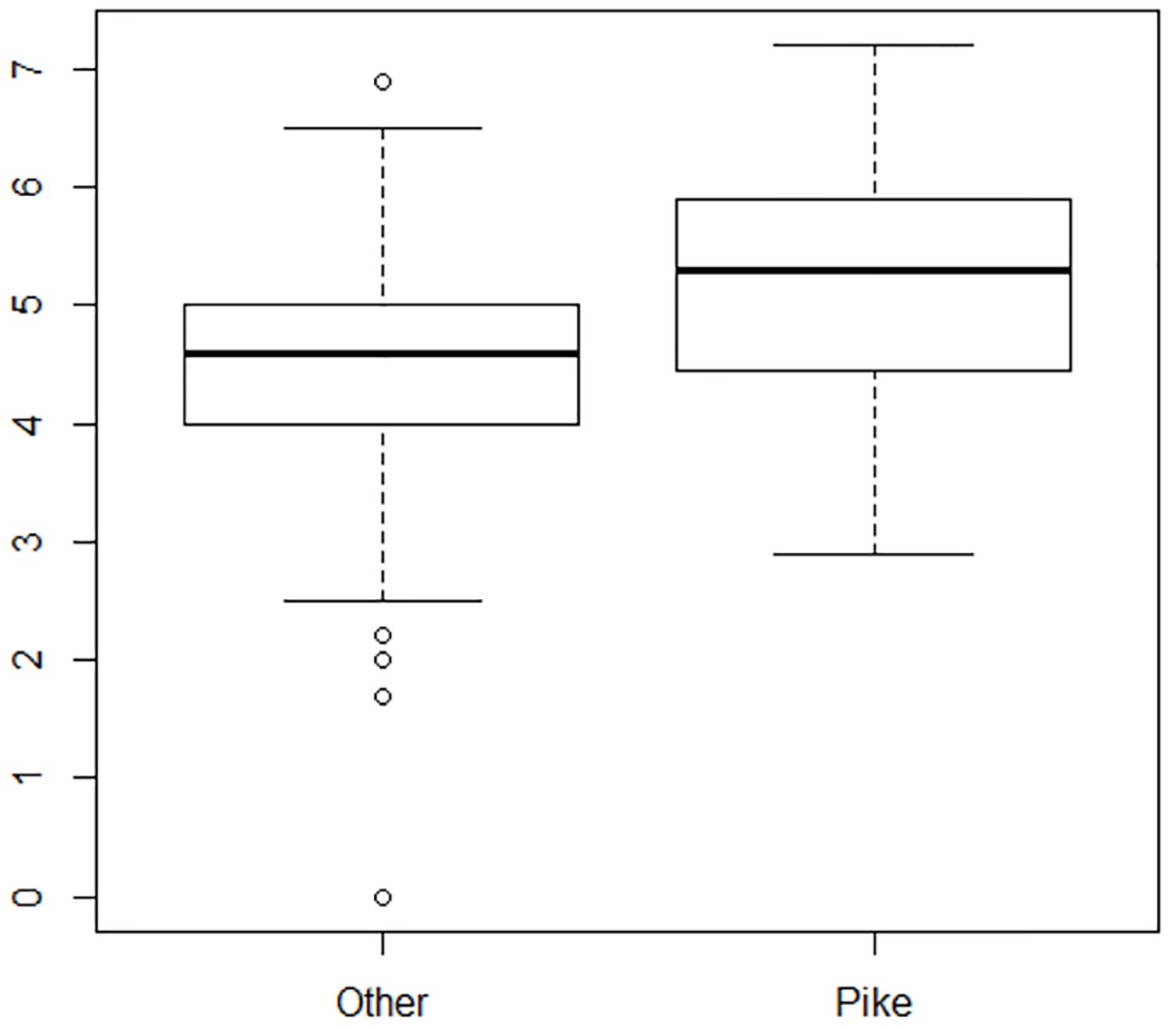
Distribution of mean scores of pike position vs. other positions. Box plots showing the distribution of mean scores once the positions free, straight, and tuck have been collapsed into the category of other.

A preliminary model that included all eight explanatory variables was fit to the data. It was determined that round number as a categorical variable with 10 levels was not a good predictor as p-values for each level ranged from 0.226 to 0.973. The first angle measurement had a p-value of 0.855 and was also deemed a poor predictor of the average score. A model that included the remaining six explanatory variables was then fit to the data. Looking at dive positions, the four categories can be collapsed into two categories: Pike and Other. This makes sense because the pike position is generally considered the most difficult, and often has a higher degree of difficulty associated with it than a tuck position for the same dive. Fig 4 shows the box plots for the average score for all the positions. Fig 5 shows the box plots once the position categories have been combined.

**Fig 6:**
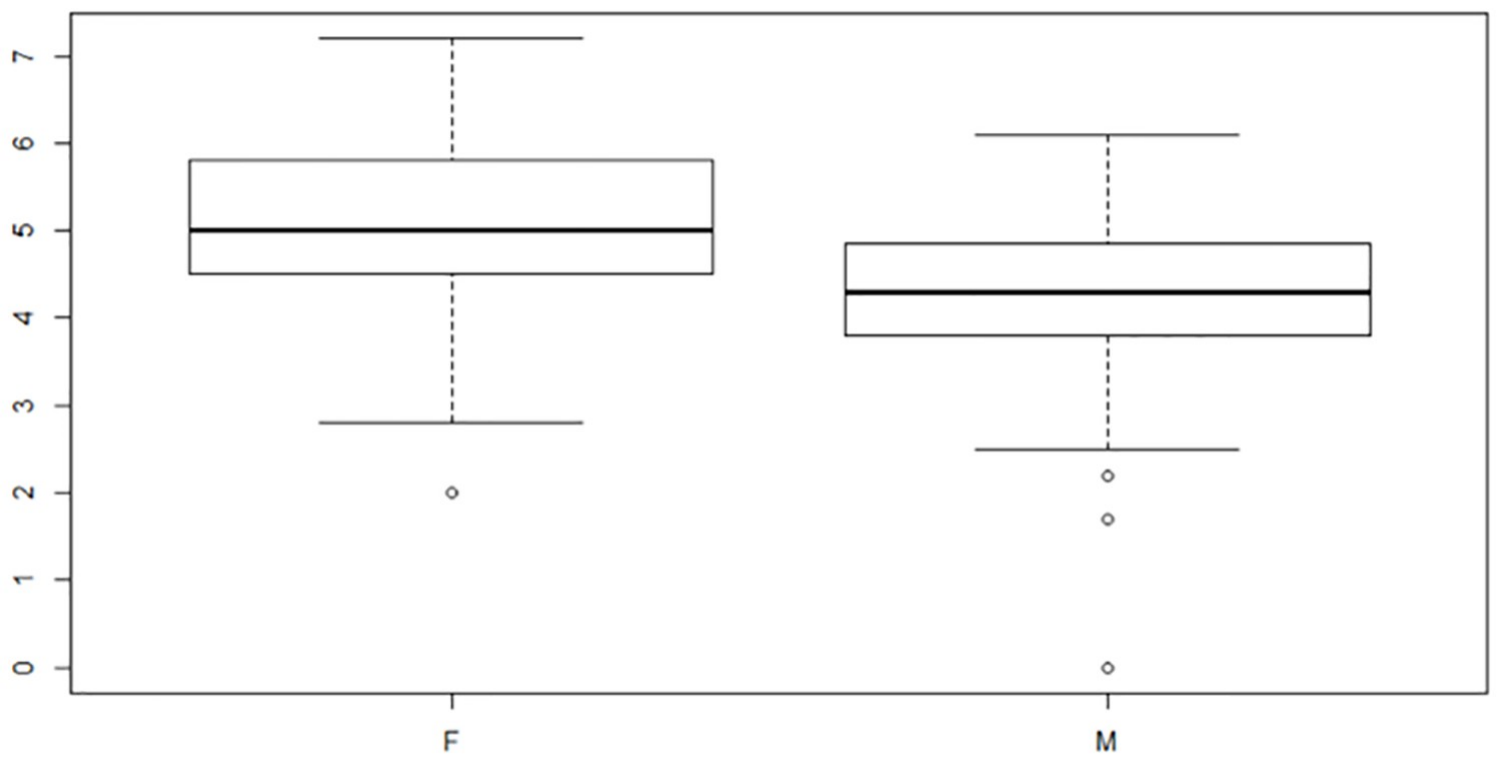
Distribution of average scores. Box plot comparing male and female average scores.

Once the diving positions had been collapsed, careful attention was paid to the order that the variables were added to the model. Gender was added first since really there are two different competitions, one for females and one for males. The difference between male and females mean scores are shown in the box plot in Fig 6.

The other variables were included in an order that prioritized the ones that took the most time to measure. Table 1 describes each variable and the order the variable was added to the model. After fitting a model with only main effects, interaction effects were then added to the model. Two of the two-way interactions had small p-values: gender and height with a p-value of 0.002 and second angle and distance with a p-value of 0.0024. The only interaction effect included in the model was gender × height since adding in second angle × distance did not greatly increase the R^2^ value. The coefficients and p-values associated with each variable are summarized in Table 2.

**Table 1:** Description of variables and the order they were added to the regression model.

Variable Name	Description	Order
Gender	Male or Female Division	1
Second Angle	Number of degrees the diver’s body is from vertical after their waist has entered the water.	2
Distance	The distance the diver is from the diving board when they break the surface of the water measured in “board marks”.	3
Height	The diver’s height in the air at the apex measured in “board marks”	4
Position	Pike or other	5
Degree of Difficulty	Degree of difficulty determined by dive	6
First Angle	Number of degrees the diver’s body is from vertical in the first three frames after entering the water.	7
Round Number	Categorical indicator variable for each round of competition	8

**Table 2:** The final model’s coefficients and p-values associated with the effects.

Variable	Significance Level	Coefficient
Intercept	<0.001	2.54
Male	0.007	1.79
Second Angle	<0.001	-0.02
Distance	<0.001	-0.08
Height	<0.001	0.22
Pike	<0.001	0.46
Degree of Difficulty	0.051	0.32
Male*Height	<0.001	-0.18

**Fig 7:**
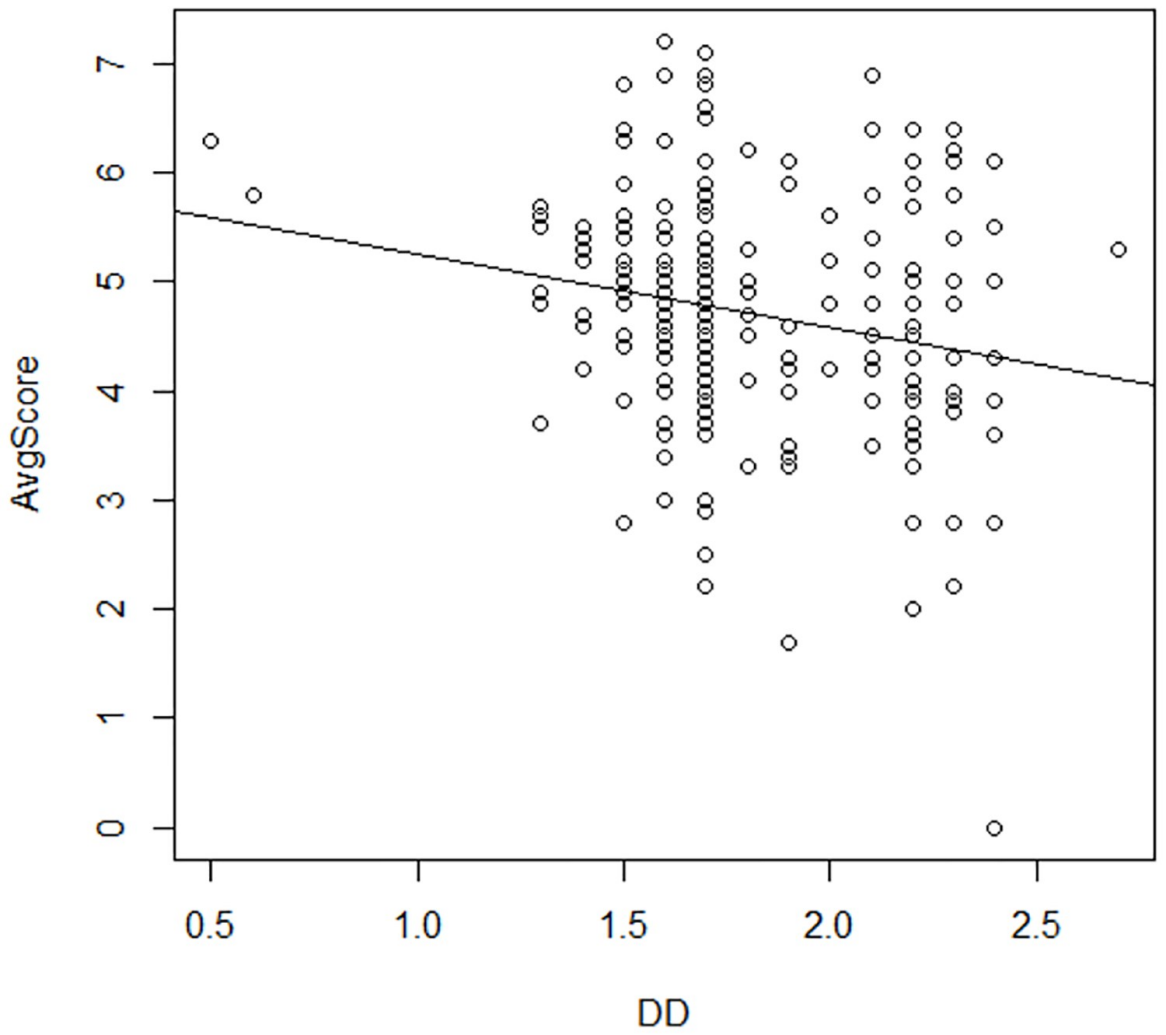
Relationship of average score to degree of difficulty. A scatter plot of Average Score vs Degree of Difficulty. Average score is on the vertical axis and degree of difficulty for each dive is on the horizontal axis. The least squares regression line has been added.

The coefficients make sense in terms of how the variables should be affecting the score. For example, as distance from the board increases, the judges’ scores decrease. This is expected, because closer distance to the board implies that the diver has more control over the dive, which indicates a better dive. Also, as the height of the dive increases the score increases. Males start out with a higher base score, but as the height of their dives increases by one mark length, their average score increases by 0.04 points whereas a female diver’s score would increase by 0.22 points. The only coefficient that defies intuition is the coefficient for the degree of difficulty. If degree of difficulty is the only regressor in the model, the coefficient is negative because more difficult dives are more difficult to execute well. This can be seen in Fig 7. Here it has flipped signs due to collinearity with other predictor variables.

Overall, the model fit the data well. The adjusted *R*^2^ value is approximately 0.46. There is no evidence of lack of fit. If we fit a model that includes only the characteristics measured from the video and gender, then the adjusted *R*^2^ value decreases to 0.42. This is not a statistically significant nor a practically important decrease, indicating that the video-based variables predict the judges’ scores well.

## Calibration of regression model with outside data

For the regional diving competition, the divers in the top three spots are eligible to move on to the state diving championship. Therefore, it is important that the top three places be correct in the sense that these divers are the ones with the greatest ability, and thus the best representatives for a given region at the state meet. Measuring the judges’ ability to obtain the correct top three rankings requires an outside measure of the divers’ ability for comparison. Fortunately, many high school divers also compete in club diving, and their results over the history of their competition are available on DiveMeets.com [27].

The DiveMeets scores are from the 2018-19 season in order to be comparable for the 2019 high school regional competition. Our main assumption is that the ability level of a diver will not change substantially over a season. Ten of the 13 female divers had participated in club diving in the past three years prior to the regional competition. Only 2 of the 12 boys had records in DiveMeets.com. This difference in the percentage of club divers among girls and boys might indicate a higher level of competition for the girls.

Table 3 shows the best score from DiveMeets.com for each of the top three girl divers in the regional competition. Their ranking in the regional competition is in the first column. Their score for the regional competition is in the second column, and their maximum score for the 2018-19 season from DiveMeets is in the third column. One can see that the divers were ranked the same in the regional competition as they were in club diving, which is evidence that the judges were judging the competition accurately. Also note that the scores from club diving were less than the scores from regional diving in all cases. This may be a reflection of more experienced judges at the club level, or it may indicate that the divers themselves were more practiced and tried more difficult dives at the regional competition due to the fact that high school regional competitions are close to the end of the club season.

**Table 3:** Places for the first three female divers in the regional competition compared to their scores on DiveMeets.

Place in Regional	Score at Regional	Best Score on DiveMeets
First	400.8	362.95
Second	397.55	328.2
Third	378.05	253.4

Of the two boys who participated in club diving, both finished in the top three. For the girls, of the 10 who participated in club diving, 6 finished in the top 8. Two divers who finished in the top 8 did not have a record of club diving in DiveMeets. Even so, the club diving scores are good independent measures of diver ability for judging the accuracy of the diving judges at the regional meet.

We compared the ranking of competitors from the regional diving meet to the rankings produced by the regression model and rankings produced from the raw measurements taken from the video. To get the rankings from the regression model, we mimicked the fashion that scores are calculated from subjective judges’ scores [5]. First, we computed the estimated total round score for each diver by using the formula round score = predicted average score × 3 × DD. We then summed the round scores to get the predicted total score. The divers were ranked based on these totals.

Obtaining ranks based on the raw measurements required adjustment to the measurements. First, we standardized each measurement by subtracting the mean and dividing by the standard deviation. Standardization puts all measurements on the same scale so that they are comparable [28]. In addition, we wanted a large positive number to represent a good dive. Thus, we subtracted the standardized value for distance from 3 so that a large distance measurement would negatively impact a diver’s ranking. The value 3 was chosen because there is a less than 1% chance that a measurement would be more than three standard deviations from the mean, assuming a Normal distribution for the measurements [28]. Figs 4–6 justify the assumption of Normality for the various measurements. We applied the same standardization and adjustment to the first and second angle measurements. We then summed the standardized values for height, distance, first angle, and second angle, as these are surrogates for judges’ scores, and multiplied by degree of difficulty to get a round total, as is done in diving competitions [5]. We summed all round totals to get the meet total and ranked the divers using the meet total. This method gives equal weight to the four characteristics measured from the footage.

The rankings determined by the regression model and the raw measurements are compared to the divers actual ranking in Tables 4 and 5. In the women’s division, the top three individuals were correctly identified by the regression model scores; however, the order of first and second place was flipped. This is not a surprise as the diver in first scored 400.80 points and the diver in second place scored 399.25 points, a very close match. Using the raw ranking method did not correctly identify the top three divers. In the men’s division, the regression method also correctly identified the top three divers, but again there was a mistake in the order, flipping the positions between the second and third place divers. A large mistake was made using the raw measurement method. Using this method, the second place diver (Diver 16) was ranked tenth. This can be attributed to Diver 16’s last dive. On this dive, the diver had a large second angle value, indicating that either the human judges could not discern a large angle of entry, or that the regression model places more emphasis on this variable than the judges do. Regardless, for both male and female divers, the regression model provided rankings that were commensurate with the actual rankings.

**Table 4:** Actual scores and rankings of all female divers compared with rankings from using the raw measurements and the regression model.

Diver	Meet Total	Raw Total	Regression Total	Ranks	Raw Ranks	Regression Ranks
3	400.80	226.52	372.34	1	2	2
8	399.25	235.65	376.22	2	1	1
4	378.05	195.43	328.34	3	4	3
14	355.20	196.14	320.04	4	3	5
11	329.80	169.10	324.52	5	8	4
13	289.85	187.99	290.75	6	5	7
7	288.03	175.94	302.59	7	7	6
2	282.30	167.55	274.56	8	10	10
5	260.75	157.65	271.85	9	11	11
10	257.05	178.76	282.33	10	6	8
9	252.10	143.28	265.90	11	12	12
6	250.30	141.76	274.81	12	13	9
12	242.05	168.84	263.99	13	9	13
1	215.50	128.38	253.54	14	14	14

**Table 5:** Actual scores and rankings of all male divers compared with rankings from using the raw measurements and the regression model.

Diver	Meet Total	Raw Total	Regression Total	Ranks	Raw Ranks	Regression Ranks
18	317.75	201.55	285.77	1	1	1
16	282.95	160.48	261.66	2	10	3
24	281.60	188.24	273.34	3	3	2
22	271.80	173.44	258.02	4	7	7
21	261.05	185.76	260.39	5	5	5
23	258.60	181.25	259.52	6	6	6
19	250.55	165.79	261.64	7	8	4
25	239.10	201.32	249.77	8	2	8
20	233.15	188.10	246.76	9	4	9
26	232.95	161.22	241.50	10	9	11
17	224.50	141.07	245.78	11	12	10
15	202.50	141.24	210.43	12	11	12

## Conclusion

Several popular sports employ panels of human judges to assign scores to competitors in the sport. It is impossible to eliminate all subjectivity when human judges are employed, as is seen by the existence of several scandals where judging bias was alleged in high-level competitions [2, 6, 7]. Video use is ubiquitous in sport, as any one who has watched any sport on television can attest. Instant replay video is often used to relive spectacular performances or review referees’ calls. In ice skating, technical panels of five judges use video to review and identify each element of a performance and the level of difficulty for that element [19]. The technical panel can use this video to verify the accuracy of elements and difficulty levels.

Diving is similar to ice skating in that there are certain technical aspects that judges expect in an exemplary performance. They are a vertical entry, a lack of splash on entry, and a minimal distance from the diving board on entry, among others [5]. In addition, certain dives are more difficult than others, and have a higher difficulty score, just as certain elements in figure skating are more difficult than others. However, unlike ice skating, technical elements of a dive are simple. Instead of the edge of a skate blade used for taking off and landing a jump, dives are measured in height and distance. Because they are simple linear measures, the technical elements of a dive can theoretically be measured from a distance in real time.

In order to build a better model that can be applied to any diving meet, we need to make use of current machine learning techniques. For example, angle of entry changes over the course of a dive. To get an accurate static measure of an instantaneous variable, we would want a computer to measure the angle of entry on every frame by recognizing the difference in color between pixels. We could then average over all these angle measurements to get a single mean angle of entry measurement. We would want to use a similar process with height in the air and distance from the diving board. This way we could get an objective measurement of maximal height and an objective measurement of distance. We believe that with these changes, it would be possible to build a real-time scoring device.

We filmed three different meets: a high school regional diving meet, a high school district meet, and an intercollegiate meet, and used the footage to measure characteristics for each dive after the meet. The regional meet is a high stakes meet for high school divers, as the top three finishers earn the right to go the state meet, while the other two meets have relatively low stakes. A linear regression model was used to model the judges scores as a function of the dive difficulty, the distance from the diving board on entry, the height of the diver at the apex of the dive, and the angle of the diver’s body at entry, in order to determine whether these features of the dive had a relationship to the judges’ scores. For the low stakes meets (data not shown), the dive features measured on video are not good predictors of the judges’ scores. This is likely due to poor accuracy of judges’ scores due to the fact that most low stakes meets employ easily accessible staff that are untrained as diving judges. Indeed, personal observation revealed judges for the low stakes meets chatting with one another or coaching a participant while other divers were competing. This is more evidence as to why supplementing the judges’ scores with data dependent video-based scores is necessary. However, in a high stakes meet, the model fit the data well enough to show that if the process of filming and measuring characteristics of a dive could be automated, then a computer could produce an objective score for each dive to supplement judges’ scores. This marriage of computer-based and subjective scores will give a good mix of the precision of the diver, which a computer can measure, and the artistic quality of a dive, which only a human can assess.
